# Anti-tumor effects of low-dose metronomic vinorelbine in combination with alpelisib in breast cancer cells

**DOI:** 10.17179/excli2022-5064

**Published:** 2023-01-13

**Authors:** Slavomir Krajnak, Jannis Patrik Trier, Pauline Friederike Heinzmann, Katharina Anic, Anne-Sophie Heimes, Amelie Loewe, Marcus Schmidt, Marco Johannes Battista, Annette Hasenburg, Walburgis Brenner

**Affiliations:** 1 Clinic for Obstetrics and Women's Health, University Medical Center of the Johannes Gutenberg-University Mainz, Mainz, Germany

**Keywords:** low-dose metronomic chemotherapy, PIK3CA mutation, vinorelbine, alpelisib, breast cancer

## Abstract

In metastatic breast cancer (MBC), *PIK3CA* mutations, activating the phosphatidylinositol 3-kinase (PI3K) signaling pathway seem to be associated with chemotherapy resistance and poor outcome. Inhibition of the PI3K signaling pathway may lead to sensitization and prevention of the development of resistance to cytotoxic drugs. The present study aimed to investigate the anti-tumor activity of low-dose vinorelbine (VRL) combined with alpelisib, an α-selective PI3K inhibitor and degrader, in breast cancer (BC) cells. Human BC cell lines MCF-7, T-47D [both hormone receptor (HR)-positive, human epidermal growth factor receptor 2 (HER2)-negative, *PIK3CA*-mutated], MDA-MB-231 and BT-549 (both triple-negative, wild-type *PIK3CA*) were exposed to a combination of low-dose VRL and alpelisib for 3 and 7 days. Cell viability was detected by the Alamar blue assay, and cell proliferation was determined by the BrdU incorporation. The effect of the substances on the p110α protein expression that is encoded by *PIK3CA *gene was investigated by Western blot. Low-dose VRL plus alpelisib showed synergistic anti-tumor effects and significantly inhibited cell viability and proliferation of MCF-7 and T-47D cells. Even lower alpelisib concentrations (10 ng/ml and 100 ng/ml) combined with low-dose metronomic VRL led to a significant reduction of cell viability of *PIK3CA*-mutated cells, and the anti-tumor activity was comparable with the effects at 1000 ng/ml alpelisib. Cell viability and proliferation of MDA-MB-231 and BT-549 cells were inhibited by VRL but not by alpelisib alone. This indicates that alpelisib did not significantly affect the cell growth of triple-negative, *PIK3CA *wild-type BC cells. The p110α expression was downregulated or not affected in *PIK3CA-*mutated cell lines, and not significantly upregulated in *PIK3CA* wild-type cell lines. In conclusion, combination of low-dose metronomic VRL and alpelisib showed synergistic anti-tumor effects and significantly inhibited the growth of HR-positive, HER2-negative, *PIK3CA*-mutated BC cells, providing a rationale for further efforts to evaluate this combination *in vivo*.

## Introduction

With an incidence of 2.3 million and a mortality of 0.7 million cases per year, female breast cancer (BC) represents the most commonly diagnosed cancer and the fifth leading cause of cancer mortality worldwide (Sung et al., 2021[42]). In the advanced/metastatic breast cancer (MBC), the goal of care is to achieve a disease chronification and preservation of the health-related quality of life (Harbeck and Gnant, 2017[20]). Despite the plethora of possible treatment options for MBC, there is still a high medical need for new treatment options that provide sufficient and long-term anti-tumor effect with a manageable toxicity profile.

The phosphatidylinositol 3-kinase (PI3K)/AKT/mammalian target of rapamycin (mTOR) signaling pathway plays one of the key roles in the regulation of cellular growth, metabolism, migration, and survival (Bilanges et al., 2019[7]; Khezri et al., 2022[23]; Li et al., 2021[27]). Genomic alterations of this pathway are detectable in many cancers, including BC (Millis et al., 2019[32]; Naeem et al., 2022[36]). It is estimated that 60-70 % of BC patients have at least one mutation in the PI3K/AKT/mTOR pathway, whereas *PIK3CA* gene, which encodes the p110α subunit of PI3K, is the most frequently mutated gene (Lee et al., 2015[26]; Millis et al., 2019[32]; Xiao et al., 2021[43]). *PIK3CA* mutation frequencies are different among BC subtypes: 34.5-48.3 %, 22.7-42.2 % and 8.3-25.0 % in HR-positive, HER2-positive, and in triple-negative BC, respectively (Martinez-Saez et al., 2020[31]; Stemke-Hale et al., 2008[41]; Xiao et al., 2021[43]). The resulting overactivation of the PI3K pathway promotes tumor growth, resistance to various systemic therapies and poor outcome (Dong et al., 2021[17]; Mosele et al., 2020[35]; Rasti et al., 2022[38]; Sobhani et al., 2018[40]; Yang et al., 2019[44]). Consequently, PI3K inhibition, especially in combination with other substances, is expected to be a new approach in the treatment of BC (Fuso et al., 2022[19]). SOLAR-1 study demonstrated a significant prolongation of progression-free survival (PFS) and 7.9-month improvement in median overall survival (OS) when alpelisib, an orally bioavailable, α-selective PI3K inhibitor and degrader, was added to fulvestrant treatment of patients with *PIK3CA*-mutated, hormone receptor (HR)-positive, human epidermal growth factor receptor 2 (HER2)-negative advanced BC (Andre et al., 2019[2], 2021[3]). Preclinical models have shown that inhibition of the PI3K signaling pathway can lead to a sensitization as well as a prevention of the development of resistance to cytotoxic drugs (Badinloo and Esmaeili-Mahani, 2014[5]; Rajput et al., 2019[37]). Metronomic chemotherapy (MCT), defined as continuous daily administration of chemotherapeutic agents at low doses, has shown promising results in MBC (Cazzaniga et al., 2019[13]; Krajnak et al., 2020[25]; Liu et al., 2017[29]; Montagna et al., 2022[33]). Based on its proven efficacy and good tolerability, MCT is increasingly perceived as a possible treatment option for MBC (Cardoso et al., 2020[10]). MCT is considered a multimodal therapy that exerts its effects via immunomodulation, inhibition of angiogenesis, and direct cytotoxic effects (Andre et al., 2017[4]; Cazzaniga et al., 2021[11]). Due to the multimodal mechanisms of action, the combination with PI3K inhibitors may represent a new promising approach in the BC treatment, preventing resistance to chemotherapy and improving prognosis. Thus, this study aimed to investigate the anti-tumor activity of low-dose metronomic vinorelbine (VRL) combined with alpelisib in various BC cell lines.

## Materials and Methods

### Cell culture and treatment

Human BC cell lines MCF-7 (HR-positive, HER2-negative with a p.E545K missense mutation in the *PIK3CA* gene), T-47D (HR-positive, HER2-negative with a p.H1047R missense mutation in the *PIK3CA* gene), MDA-MB-231 and BT-549 (both triple-negative, wild-type *PIK3CA*), were all obtained from American Type Culture Collection (Rockville, MD, USA). BC cells were cultured in RPMI 1640 culture medium (Thermo Fisher Scientific, Waltham, MA, USA), containing 10 % fetal calf serum and 1 % penicillin-streptomycin. Cells were incubated in a moistened atmosphere at 37 °C and 5 % CO_2_. For all functional assays, cells were detached by trypsin-ethylenediaminetetra-acetic acid (Sigma Aldrich, St. Louis, MO, USA) and seeded in 96-well plates (Greiner Bio-One, Frickenhausen, Germany) at 3,000 cells/well (passage 10/semiconfluence). VRL (Navirel®; Medac, Wedel, Germany), (stock solution 10 mg/ml) diluted in Aqua dest., was added at levels corresponding to the concentration in serum of metronomically treated patients (0.63-5 ng/ml VRL), *i.e.* at much lower concentrations compared to maximum tolerated dose of conventional chemotherapy (Bocci and Kerbel, 2016[8]; Briasoulis et al., 2013[9]). Alpelisib (Piqray®, Novartis Pharma AG, Basel, Switzerland) (stock solution 2 mg/ml), diluted in 100 % dimethyl sulfoxide (DMSO) (Carl Roth GmbH, Karlsruhe, Germany), was added at concentrations equivalent to serum concentration in patients receiving an approved dose of 300 mg/day (500-1000 ng/ml) (Juric et al., 2018[22]). In addition, in view of a possible reduction of side effects *in vivo*, alpelisib was tested also at lower concentrations (10 and 100 ng/ml). The treatment with both substances was performed continuously for 3 and 7 days to simulate the metronomic dosing schedule. 

### Cell viability assay

Cell viability was measured using the Alamar blue assay kit (Thermo Fisher Scientific). After 3 and 7 days of treatment, 100 µl/well Alamar blue Cell Viability Reagent solution [(1:10 in Dulbecco's Phosphate-Buffered Solution (DPBS)] was added to the cell cultures and incubated for 4 hours at 37 °C and 5 % CO_2_ in aluminum foil due to the photosensitivity of the redox dye for fluorescence measurements. The absorbance was measured at 590 nm (650 nm reference wavelength) using a GloMax® multi detection system microplate reader (Anthos Labtec Instruments, Cambridge, UK).

### Cell proliferation assay

Cell proliferation was detected by the Bromdeoxyuridine (BrdU) incorporation kit (Roche, Basel, Switzerland). 10 μl/well BrdU labeling solution (1:100 in DPBS) was added to the cells and incubated for 3 hours at 37 °C and 5 % CO_2_. Afterwards supernatants were discarded and 200 ml/well Fix-Denat solution and 100 μl/well anti-BrdU antibody solution (1:100 in antibody dilution solution) were added. After incubation for 60 min at room temperature the absorbance was measured at 450 nm (690 nm reference wavelength) using a GloMax® multi detection system microplate reader (Anthos Labtec Instruments).

### Western blot

To prepare protein extracts from cell culture, tumor cells were seeded on 143 mm^2^ cell culture plates. The cell number was chosen according to the proliferation rate so that the culture dishes were semiconfluent at the time of protein extraction. One day after seeding, the cells were treated for 7 days. For protein extraction, cells were washed with DPBS and mechanically removed with a cell scraper. The solution was centrifuged at 1200 rpm for 5 minutes. The supernatant was discarded and the pellet was dissolved with up to 1 ml of lysis buffer. The solution was transferred into a 2 ml reaction tube and placed on ice. After incubation for 30 min on ice the samples were centrifuged for 10 min at 14000 g. The supernatant was transferred to a new tube and stored at -20 °C. The BCA Protein assay kit (Thermo Fisher Scientific) was used to determine the protein concentration of the extracts. Equal amounts of protein (12.5 μg per lane) were separated by size using sodium dodecyl sulfate polyacrylamide gel electrophoresis (SDS-PAGE) with 10 % polyacrylamide gels. Gels were transferred on polyvinylidene difluoride (PVDF) membrane by semi-dry blotting. Membranes were blocked for 1 hour according to the antibody manufacturer's instructions and then incubated with a primary antibody in blocking solution overnight at 4 °C on a roll mixer. The monoclonal antibody against PI3K p110α (Cell Signaling Technology, Danvers, MA, USA) was used at a dilution of 1:500. β-actin antibody (Sigma Aldrich) was employed at a dilution of 1:2000. After washing, the membranes were incubated with HRP-linked secondary antibody (Agilent, Santa Clara, CA, USA) at a dilution of 1:4000 (PI3K p110α Western blot) or 1:2000 (β-actin Western blot) for 1 hour at room temperature, and after washing the bound antibodies were visualized by adding of an enhanced chemiluminescent solution (PerkinElmer Inc, Waltham, MA, USA) and detected in a chemiluminescence detector (ProteinSimple, San Jose, CA, USA). For quantification bands were quantified by densitometry evaluation using a computer-based pixel counting system (AlphaView, ProteinSimple). These values were referenced to the corresponding β-actin values of the same membrane as a loading control.

### Statistical analysis

All experiments were performed in triplicates and repeated three times. The absorbance in Alamar blue and BrdU assay for the untreated control group was regarded as 100 % cell viability and 100 % cell proliferation, respectively. The results of absorbance for the treated groups were indicated as the mean ± standard deviation of three separate experiments and displayed as percentages relative to that of the control group. Student's* t-*test (Microsoft Excel 2013 v15.0; Microsoft Corporation, Redmond, WA, USA) was used to evaluate the statistical significance of the results. All p-values represented two-sided tests and statistical significance was assumed at a value of p<0.05. To quantify the effects of combination treatment, the combination index (CI)-isobologram equation based on the Chou-Talalay method was used (Chou, 2006[14]). Based on the experimentally determined dose-response curves, the inhibitory concentrations (IC)_50_ and IC_80_ were calculated by interpolation and used as a reference for further investigation of any synergistic effects according to the CI method. The CI was calculated according to the formula shown in formula 1 and was interpreted as follows: CI<1, synergism; CI=1, additive effect, and CI>1, antagonism (range ± 5 %). The graphical presentation was performed as a classical isobologram with isoboles of the IC_50_ or IC_80_ and presentation of the corresponding CI values in relation to the corresponding isoboles within the diagram.


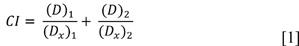


where CI = combination index (CI). (D)_1_ and (D)_2_: required concentrations of the active compounds in combination to achieve the defined effect.(D_x_)_1_ and (D_x_)_2_: required concentrations of each active compound considered individually to achieve defined effect. 

## Results

### Effects of low-dose vinorelbine plus alpelisib on cell viability

**Figure 1:**
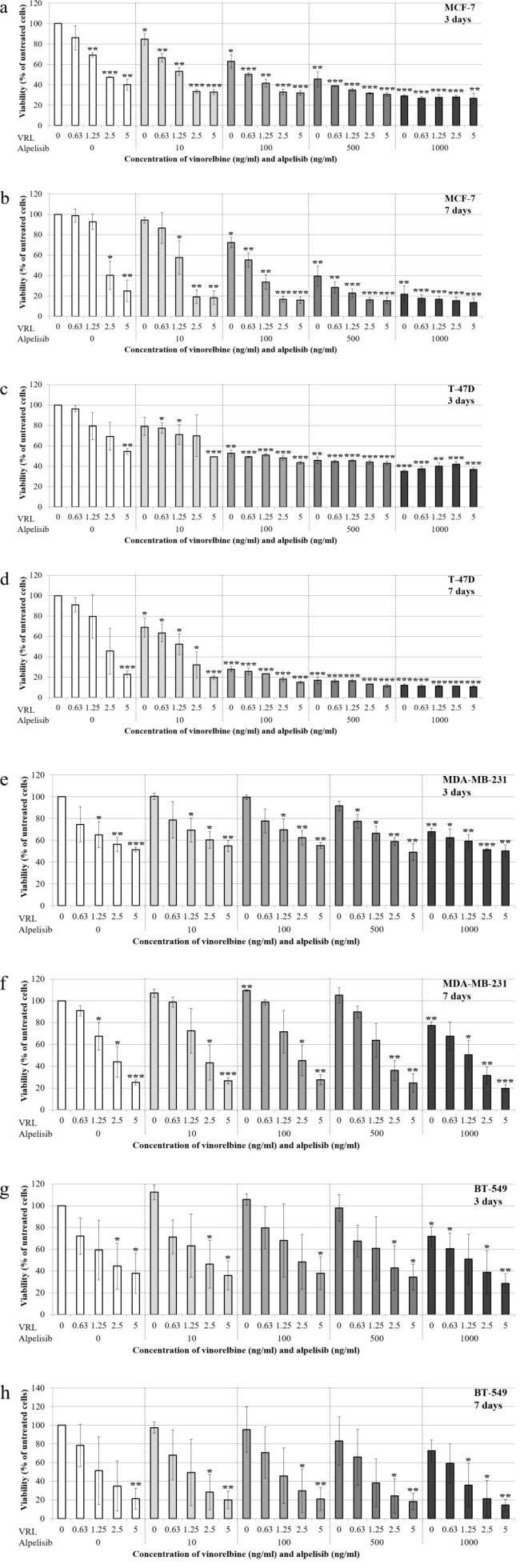
Effects of low-dose vinorelbine plus alpelisib on cell viability of the MCF-7 (a, b), T-47D (c, d), MDA-MB-231 (e, f) and BT-549 (g, h) cell line. Alamar blue assay was used to measure cell viability after 3 and 7 days of treatment with low-dose vinorelbine plus alpelisib. The results are shown as the mean ± standard deviation of three separate experiments. Statistical significance was assumed at *p<0.05, **p<0.01 and ***p<0.001.

In both HR-positive, *PIK3CA*-mutated cell lines, the combination of low-dose VRL and alpelisib decreased cell viability with increasing concentrations after both 3 and 7 days of treatment (Figure 1a-1d(Fig. 1)). Even the combination of 0.63 ng/ml VRL and 10 ng/ml alpelisib significantly reduced cell viability of MCF-7 cells by 33.5 % (p=0.005) (Figure 1a(Fig. 1)) and T-47D cells by 22.8 % (p=0.016) (Figure 1c(Fig. 1)) after 3 days. At 2.5 ng/ml VRL plus 10 ng/ml alpelisib, cell viability of MCF-7 was 33.4 % after 3 days (p<0.001) and 19.1 % after 7 days (p=0.002), and decreased slightly to 26.8 % (p=0.002) and 13.5 % (p<0.001) at the highest concentrations (Figure 1a, 1b(Fig. 1)). Similar response of VRL plus alpelisib on cell viability was observed in T-47D cells. At 2.5 ng/ml VRL plus 100 ng/ml alpelisib, cell viability of T-47D was 48.0 % after 3 days (p<0.001) and 18.1 % after 7 days (p<0.001), and decreased slightly to 36.8 % (p<0.001) and 10.6 % (p<0.001) at the highest concentrations (Figure 1c, 1d(Fig. 1)).

In the triple-negative, *PIK3CA* wild-type cell lines, the combination of low-dose VRL and alpelisib reduced cell viability with increasing concentrations after both 3 and 7 days of treatment (Figure 1e-1h(Fig. 1)). Concentrations of 2.5 ng/ml VRL plus 10 ng/ml alpelisib significantly decreased cell viability of MDA-MB-231 cells by 56.9 % (p=0.026) (Figure 1f(Fig. 1)) and BT-549 cells by 71.7 % (p=0.023) (Figure 1h(Fig. 1)) after 7 days. The strongest effects with the lowest cell viability of MDA-MB-231 cells (19.7 %, p<0.001) and BT-549 cells (14.4 %, p=0.002) were observed at 5 ng/ml VRL and 1000 ng/ml alpelisib after 7 days of treatment (Figure 1f, 1h(Fig. 1)). In contrast to HR-positive cells with a *PIK3CA* mutation, the reduction of cell viability was not or only marginally affected by alpelisib alone. Concretely, alpelisib in concentrations below 1000 ng/ml did not significantly affect cell viability of the triple-negative *PIK3CA* wild-type cells tested. 

### Effects of dimethyl sulfoxide on cell viability

**Figure 2:**
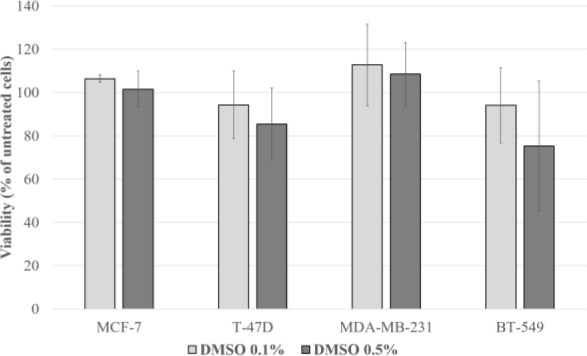
Effects of DMSO on cell viability of MCF-7, T-47D, MDA-MB-231 and BT-549 cells after 7 days of treatment at a concentration corresponding to the highest alpelisib concentration (0.5 % DMSO≙1000 ng/ml alpelisib) and in a concentration corresponding to a lower alpelisib concentration (0.1 % DMSO≙100-500 ng/ml alpelisib). The results are shown as the mean ± standard deviation of three separate experiments. Statistical significance was assumed at *p<0.05, **p<0.01 and ***p<0.001.

The effects of DMSO on cell viability after 7 days of treatment were assessed at a concentration corresponding to the highest alpelisib concentration (0.5 % DMSO≙1000 ng/ml alpelisib) and in a concentration corresponding to a lower alpelisib concentration (0.1 % DMSO≙100-500 ng/ml alpelisib). In all cell lines tested DMSO at concentrations 0.1-0.5 % did not significantly affect the cell viability (Figure 2(Fig. 2)). However, the viability of BT-549 cells decreased by 24.7 % (p=0.319) when treated with 0.5 % DMSO, and thus this cell line was not used for further cell culture experiments due to potential confounding of the results.

### Effects of low-dose vinorelbine plus alpelisib on cell proliferation

**Figure 3:**
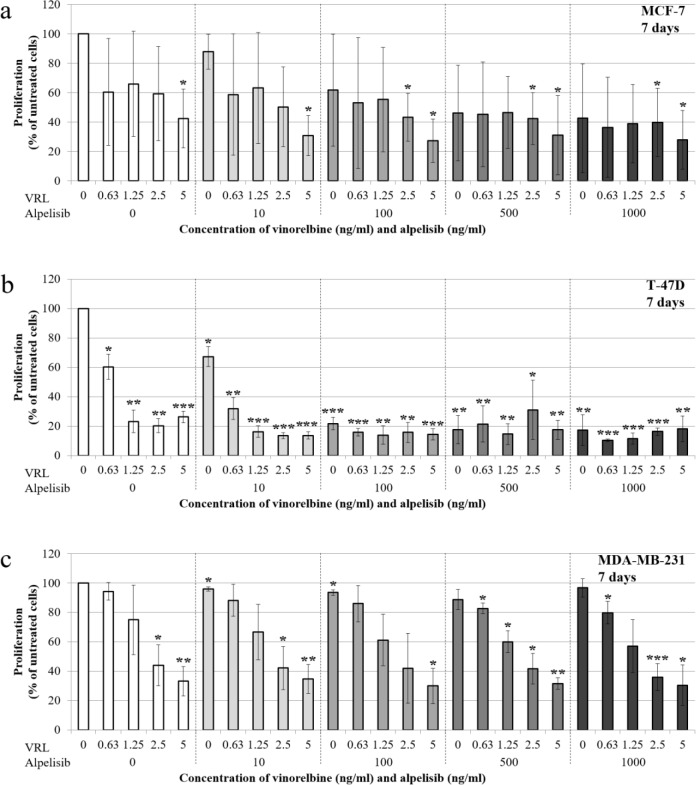
Effects of low-dose vinorelbine plus alpelisib on cell proliferation of the MCF-7 (a), T-47D (b) and MDA-MB-231 (c) cell line. BrdU incorporation was used to measure cell proliferation after 7 days of treatment with low-dose vinorelbine plus alpelisib. The results are shown as the mean ± standard deviation of three separate experiments. Statistical significance was assumed at *p<0.05, **p<0.01 and ***p<0.001.

In both HR-positive, *PIK3CA*-mutated cell lines, the combination of low-dose VRL and alpelisib decreased cell proliferation with increasing concentrations after 7 days of treatment (Figure 3a, 3b(Fig. 3)). Similar to the cell viability results, the reduction of cell proliferation was detectable also with single agents. Combination of 2.5 ng/ml VRL and 10 ng/ml alpelisib significantly reduced cell proliferation of MCF-7 by 32.9 % (p=0.004) and the highest concentrations reduced cell proliferation by 65.1 % (p=0.005) after 7 days of treatment (Figure 3a(Fig. 3)). In T-47D cells, even the lowest concentration of 0.63 ng/ml VRL plus 10 ng/ml alpelisib significantly decreased cell proliferation by 68.1 % (p=0.004) after 7 days of treatment (Figure 3b(Fig. 3)). At 2.5 ng/ml VRL plus 10 ng/ml alpelisib, cell proliferation was 13.5 % (p<0.001) and varied only marginally with the minimal value of 10.3 % (p<0.001) at higher concentrations.

In the triple-negative, *PIK3CA* wild-type MDA-MB-231 cells, the combination of low-dose VRL and alpelisib reduced cell proliferation with increasing concentrations. Combination of 2.5 ng/ml VRL and 10 ng/ml alpelisib significantly decreased cell proliferation of MDA-MB-231 cells by 57.8 % (p=0.021) after 7 days of treatment (Figure 3c(Fig. 3)), whereby increasing concentrations of alpelisib did not show further anti-tumor activity. The lowest cell proliferation of around 30.0 % was achieved at 5 ng/ml VRL and at alpelisib concentrations of above 100 ng/ml (p=0.010). This indicates that alpelisib did not significantly affect the cell proliferation of triple-negative, *PIK3CA *wild-type MDA-MB-231 cells.

### Synergistic effects of low-dose vinorelbine plus alpelisib on cell viability and proliferation in HR-positive, PIK3CA-mutated cell lines

**Figure 4:**
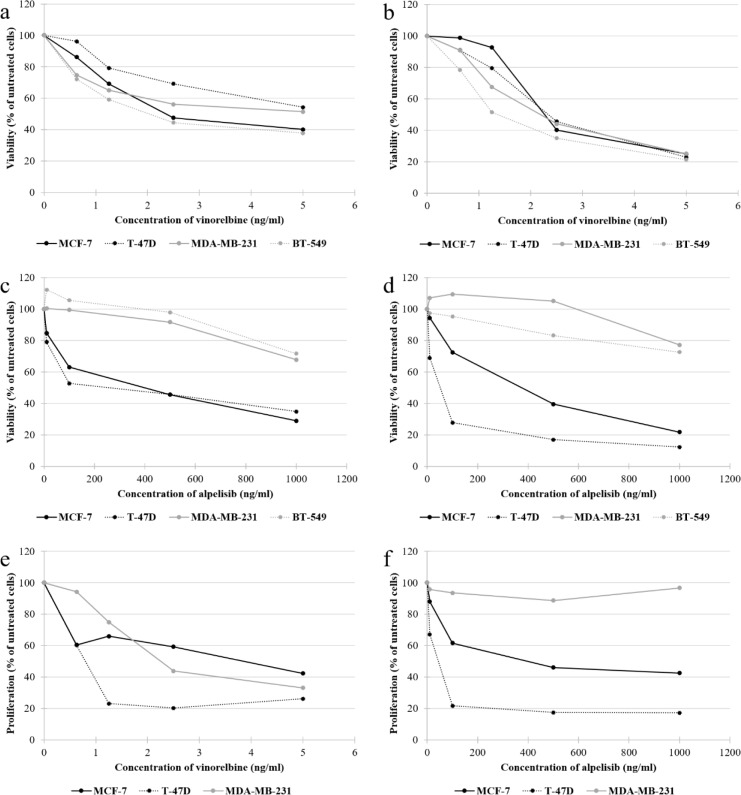
Dose-response curves of vinorelbine and alpelisib regarding cell viability: vinorelbine (a, b), alpelisib (c, d) after 3 and 7 days of treatment and regarding cell proliferation: vinorelbine (e), alpelisib (f) after 7 days of treatment. The results are shown as the mean of three separate experiments. The standard deviation results are shown in Figure 1 and Figure 3, respectively, and have been omitted from this figure for clarity.

**Figure 5:**
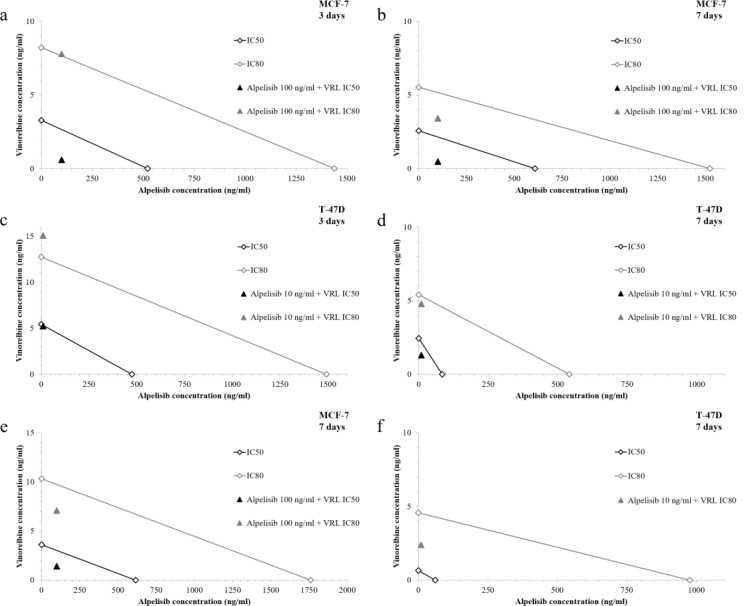
Combination index-isobologram for cell viability of the MCF-7 (a, b) and T-47D (c, d) cell line after 3 and 7 days of treatment and for cell proliferation of the MCF-7 (e) and T-47D (f) cell line after 7 days of treatment with low-dose vinorelbine plus alpelisib. The inhibitory concentrations (IC)_50_ and IC_80_ were calculated by interpolation and used as a reference. The combination index (CI) was calculated according to the formula shown in formula [1] and was interpreted as follows: CI<1, synergism; CI=1, additive effect, and CI>1, antagonism (range ± 5 %). The graphical presentation was performed as classical isobologram with isoboles of the IC_50_ or IC_80_ and presentation of the corresponding CI values in relation to the corresponding isoboles within the diagram.

To determine the synergistic effects of low-dose VRL plus alpelisib on cell viability according to the isobole method and to calculate the CI in MCF-7 cells, concentration of 100 ng/ml alpelisib was used. For the application of the isobole method the IC_50_ and IC_80_ were calculated and dose-response curves for single compounds were generated (Figure 4(Fig. 4)). After 3 days of treatment, 0.59 ng/ml and 7.81 ng/ml VRL was required to achieve the IC_50_ and IC_80_, respectively (Figure 5a(Fig. 5)). The CI was 0.372 for the IC_50_ and 1.018 for the IC_80_ value, so conceptually synergistic and additive effects could be assumed, respectively. The synergistic effects were stronger after 7 days of treatment. The IC_50_ value was reached at 0.48 ng/ml VRL (CI=0.351) and the IC_80_ value at 3.43 ng/ml VRL (CI=0.685) (Figure 5b(Fig. 5)). For the calculation of the CI in T-47D cells, concentration of 10 ng/ml alpelisib was used to achieve interpretable data. After 3 days of treatment, 5.23 ng/ml and 15.08 ng/ml VRL was required to achieve the IC_50_ (CI=0.987) and IC_80_ (CI=1.190) value (Figure 5c(Fig. 5)). The synergistic effects were evident after 7 days of treatment. The IC_50_ was reached at 1.29 ng/ml VRL (CI=0.648) and the IC_80_ at 4.79 ng/ml VRL (CI=0.906) (Figure 5d(Fig. 5)).

To determine the synergistic effects of low-dose VRL plus alpelisib on cell proliferation according to the isobole method and to calculate CI in MCF-7 cells, concentration of 100 ng/ml alpelisib was used. After 7 days of treatment, 1.41 ng/ml and 7.10 ng/ml VRL was required to achieve the IC_50_ and IC_80_ value, and the CI of 0.554 and 0.744 revealed synergistic effects of the agents, respectively (Figure 5e(Fig. 5)). In T-47D cells, the isobole method of calculating CI with 10 ng/ml alpelisib showed that 2.39 ng/ml VRL was required to reach IC_80_ value after 7 days of treatment (Figure 5f(Fig. 5)). The CI was 0.533, suggesting synergistic effects. The calculation of IC_50_ seemed implausible and was not performed due to the strong reduction of cell proliferation already at low concentrations.

### Effects of low-dose vinorelbine plus alpelisib on p110α expression

**Figure 6:**
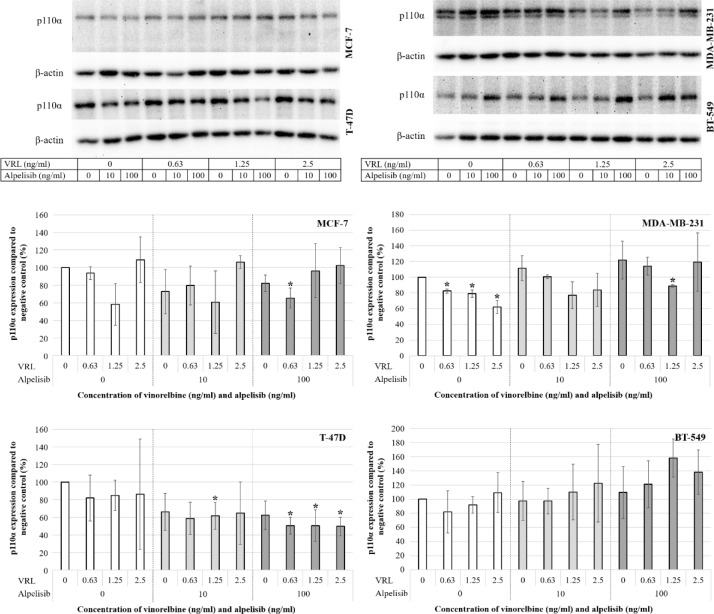
Western blot analyses of the p110α expression in the *PIK3CA*-mutated cell lines MCF-7 and T-47D (a) and the *PIK3CA* wild-type cell lines MDA-MB-231 and BT-549 (b) after 7 days of treatment with low-dose vinorelbine plus alpelisib. 12.5 µg protein was separated in a 10 % polyacrylamide gel. The order of the concentrations of substances differs between the bars and bands.

To further examine the effects of low-dose VRL and alpelisib in the *PIK3CA*-mutated and *PIK3CA* wild-type cell lines, we analyzed the expression of the p110α protein that is encoded by the *PIK3CA* gene. In the MCF-7 cell line, the p110α expression was not significantly affected at most concentrations tested by VRL, alpelisib or combination of both substances (Figure 6a(Fig. 6)). In T-47D cells, the p110α expression was downregulated by alpelisib (Figure 6a(Fig. 6)). In both triple-negative, *PIK3CA *wild-type cell lines MDA-MB-231 and BT-549, an increase of the p110α expression induced by alpelisib was detected (Figure 6b(Fig. 6)). VRL at a concentration of 5 ng/ml and alpelisib at concentrations of 500 ng/ml and 1000 ng/ml led to a significant reduction in the number of cells tested. This resulted in a substantial reduced protein concentration in the cell lysate during protein extraction, making it impossible to obtain the amount of protein required for a Western blot. Therefore, these concentrations of the agents are missing in the Western blot analyses.

See also the Supplementary data.

## Discussion

In this study, the combination of low-dose metronomic VRL and alpelisib revealed a significant reduction of cell viability and proliferation with synergistic anti-tumor effects in HR-positive, HER2-negative, *PIK3CA*-mutated BC cell lines. The growth of triple-negative, *PIK3CA* wild-type cell lines was significantly inhibited by VRL but not by alpelisib alone. These results confirm the hypothesis that PI3K inhibitors may potentiate cytotoxic activity of anti-microtubule agents in *PIK3CA*-mutated BC cell lines, as previously described (Badinloo and Esmaeili-Mahani, 2014[5]; Morgillo et al., 2017[34]; Rajput et al., 2019[37]). Taselisib, a selective inhibitor of class I PI3Kα, δ-, and γ-isoforms, and ipatasertib, an AKT inhibitor, plus anti-microtubule chemotherapy showed significant synergism in terms of antiproliferative, pro-apoptotic, and anti-metastatic effects in *PIK3CA*-mutated BC cells (Morgillo et al., 2017[34]). In particular, MCF-7 (HR-positive, HER2-negative), BT474 (HR-positive, HER2-positive), KPL-4 (HR-negative, HER2-positive), and SUM159 (triple-negative) BC cell lines with* PIK3CA* mutation were treated for 3 days with taselisib or ipatasertib and VRL, eribulin, and paclitaxel in concentrations of IC_50_. Combined treatment with taselisib and anti-microtubule agents exerted a strong reduction of cell viability with synergistic effects and a significant increase of apoptotic cells in all cell lines tested compared to single treatment. In addition, the combination of taselisib or ipatasertib and eribulin significantly inhibited motility and migration of SUM159 mesenchymal cells compared to single-agent treatment. Here, by way of comparison, we intended to simulate the metronomic dosing regimen by continuously treating the cells with low-dose chemotherapeutic agent over an extended period of time and observed similar results with combination of alpelisib and VRL. In both *PIK3CA*-mutated BC cell lines, we observed a CI<1, assuming synergistic effects of low-dose VRL and alpelisib. The synergistic cytotoxic effects were after 7 days of treatment even stronger compared to 3 days of treatment, suggesting a favorable impact of continuous long-term administration of VRL and alpelisib. In the Western blot analyses, the p110α expression was not affected or downregulated in the *PIK3CA-*mutated cell lines MCF-7 and T-47D, respectively, and not significantly upregulated in the *PIK3CA* wild-type cell lines MDA-MB-231 and BT-549. It may be assumed that the p110α expression did not show any clear dependance on alpelisib at concentrations tested in HR-positive, HER2-negative cell lines with an overactivation of p110α due to the *PIK3CA* mutation. In contrast, the triple-negative *PIK3CA* wild-type cell lines showed regulatory mechanisms associated with upregulation of the non-overactivated protein as a response to treatment with alpelisib.

Serum concentration achieved in patients taking 300 mg alpelisib once daily on a regular basis averages 1000 ng/ml (Juric et al., 2018[22]). Here, we showed that even lower alpelisib concentrations (10 ng/ml and 100 ng/ml) combined with low-dose metronomic VRL led to a significant reduction of cell viability of *PIK3CA*-mutated cells, and the anti-tumor activity was comparable with the effects at 1000 ng/ml alpelisib. This indicates that above a certain concentration of VRL and alpelisib, no significant potentiation of the anti-tumor effect could be observed. The combination of the agents in lower doses may reduce side effects, so the efficacy and tolerability will be further evaluated *in vivo*.

Hyperactivation of PI3K/AKT/mTOR pathway has also been associated with triple-negative breast cancer (TNBC) via loss of phosphatase and tensin homolog (PTEN), a direct counterpart of PI3K (Lopez-Knowles et al., 2010[30]). Therefore, Rajput and colleagues (2019[37]) assessed the combinatory effect of eribulin and buparlisib, a pan-class I PI3K inhibitor, in TNBC cell lines and patient-derived xenograft (PDX) models. In the BT-549, HCC1806, and MBA-MB-231 TNBC cell lines as well as WHIM3 and WHIM12 PDX derived cell lines with loss of PTEN, reduction of cancer stem cell population and synergistic cytotoxic effects between eribulin and buparlisib were observed. In the randomized phase III trials BELLE-2 and BELLE-3, treatment with buparlisib and fulvestrant demonstrated efficacy with significant prolongation of PFS compared to fulvestrant alone in pretreated HR-positive, HER2-negative MBC patients. However, the unfavorable toxicity profile of buparlisib including elevation of liver enzymes, hyperglycemia, depression, and rash led to terminate further development of the agent (Baselga et al., 2017[6]; Di Leo et al., 2018[16]). In the randomized phase III study SANDPIPER, taselisib in combination with fulvestrant also met its primary endpoint and underlined the efficacy in HR-positive, HER2-negative, *PIK3CA*-mutated MBC patients. However, the combination did not demonstrate clinical utility given its safety profile and modest clinical benefit (Dent et al., 2021[15]). Combination of fulvestrant and alpelisib (300 mg once daily) versus placebo was evaluated in the randomized phase III study SOLAR-1. The addition of alpelisib demonstrated a significant 5.3-month improvement in median PFS and a 7.9-month improvement in median OS in HR-positive, HER2-negative, *PIK3CA*-mutated MBC patients. In the cohort without *PIK3CA*-mutated BC, there was no significant difference in the median PFS (7.4 months versus 5.6 months), suggesting that the *PIK3CA* gene may represent a predictive biomarker for alpelisib activity in this population (Andre et al., 2019[2]). In the overall population, the most frequent adverse events of grade 3 or 4 were hyperglycemia (36.6 % versus 0.7 %) and rash (9.9 % versus 0.3 %). In the SOLAR-1 *PIK3CA*-mutated cohort, only 5.9 % of patients had previously received cyclin-dependent kinase 4/6 inhibitor (CDK4/6i). Since endocrine therapy combined with CDK4/6i has become the standard first-line treatment for HR-positive, HER2-negative MBC, a phase II trial, BYLieve (NCT03056755), was conducted to evaluate alpelisib plus fulvestrant in MBC patients who had progressed on immediate prior CDK4/6i plus aromatase inhibitor. In a cohort A, alpelisib plus fulvestrant met its primary endpoint, with 50.4 % of patients alive without progression after 6 months of treatment (Rugo et al., 2021[39]).

Considering that clinical trials evaluating the combination of alpelisib plus endocrine therapy have shown favorable efficacy and manageable toxicity profile in HR-positive, HER2-negative, *PIK3CA*-mutated MBC patients, there is a promising approach to investigate alpelisib with chemotherapeutic agents as well. VRL, an orally available anti-microtubule agent, represents a standard treatment option in MBC (Aapro et al., 2019[1]; Huang et al., 2020[21]). In addition, VRL is one of the most commonly used agents for MCT with proven efficacy and excellent safety profile (Cazzaniga et al., 2019[13]; Liu et al., 2021[28]). Based on the growing body of evidence, MCT can be considered as a suitable treatment option in selected MBC patients. Especially patients with HR-positive, HER2-negative metastatic disease resistant to endocrine-based therapy and not requiring rapid tumor response are generally suitable for MCT (Cazzaniga et al., 2019[12]; Krajnak et al., 2022[24]). Alpelisib is an approved, orally bioavailable, α-selective inhibitor of PI3K for use in combination with fulvestrant to treat HR-positive, HER2-negative, *PIK3CA*-mutated MBC patients after endocrine-based therapy (Andre et al., 2019[2]). Alpelisib targets the two most common *PIK3CA* mutations and is 50 times more potent against PI3Kα than other isoforms (Fritsch et al., 2014[18]). As there is evidence that inhibition of the PI3K pathway may prevent resistance to chemotherapy and potentiate its efficacy, the combination of PI3K inhibitors with MCT affecting angiogenesis, immune response, and tumor cells via direct cytotoxicity may represent a new promising approach for the treatment of MBC, aiming to achieve synergistic effects, overcome drug resistance, or decrease the drug dose and toxicities. The fact that both agents are specifically indicated for the treatment of HR-positive, HER2-negative MBC after CDK4/6i pretreatment also supported the investigation of the substances in combination.

In this study, HR-positive, HER2-negative cell lines with a *PIK3CA* mutation and triple-negative cell lines without a *PIK3CA* mutation were analyzed, so we cannot clearly state whether the significant reduction in the cell viability and proliferation shown depended on the *PIK3CA* mutation alone regardless of HR and HER2 status. Although we hypothesize that the *PIK3CA* mutation was primarily responsible for the synergistic effects and the effect of alpelisib shown, we consider the evaluation of HR-positive, HER2-negative cell lines without *PIK3CA* mutation as well as the evaluation of triple-negative cell lines with a *PIK3CA* mutation to be of particular importance.

In conclusion, the present work revealed a significant reduction of cell viability and proliferation with synergistic effects of low-dose metronomic VRL and alpelisib in HR-positive, HER2-negative, *PIK3CA*-mutated BC cell lines. In addition, we showed that even lower doses of alpelisib in combination with low-dose VRL result in significant anti-tumor effects, providing a rationale for further efforts to evaluate this combination *in vivo* with the intent of improving the toxicity profile.

## Declaration

### Conflict of interest

SK received speaker honoraria from Roche Pharma AG and Novartis Pharma GmbH Germany, research funding from Novartis Pharma GmbH Germany and travel reimbursement from PharmaMar and Novartis Pharma GmbH Germany.

KA received speaker honoraria from Clovis Oncology, MSD und AstraZeneca.

ASH received speaker honoraria from Pfizer Pharma GmbH and honoraria from Medupdate GmbH.

MS received honoraria for speaker or consultancy role from AMGEN, AstraZeneca, Eisai, Lilly, Myelo Therapeutics, Novartis, Pantarhei Bioscience, Pfizer, and Roche. He received research funding from AstraZeneca, BioNTech, Eisai, Genentech, Myelo Therapeutics, Novartis, Pantarhei Bioscience, Pfizer, Pierre-Fabre, and Roche Pharma AG. He received travel reimbursement from Pfizer and Roche. In addition, MS has a patent for EP 2390370 B1 issued and a patent for EP 2951317 B1issued.

MJB received honoraria and expenses from Astra Zeneca, Clovis Oncology, GSK, MSD, Pharma Mar, Roche Pharma AG and Tesaro Bio Germany GmbH. He is consultant to Eisai, GSK, MSD, Pharma Mar, Roche Pharma AG and Tesaro Bio Germany GmbH. He received funded research from AstraZeneca, Clovis Oncology, MSD and Novartis.

AH received honoraria from AstraZeneca, Celegen, MedConcept Gm, Med update GmbH, Medicultus, Pfizer, Promedicis GmbH, Pierre Fabre, Softconsult, Roche Pharma AG, Streamedup!GmbH and Tesaro Bio Germany GmbH. She is a member of the advisory board of PharmaMar, Promedicis GmbH, Pierre Fabre Pharma GmbH, Roche Pharma AG and Tesaro Bio Germany GmbH. She received research funding from Celgene. 

All remaining authors declare that there are no conflicts of interest regarding the publication of this manuscript.

### Authors' contributions

Conceptualization: SK, MJB and WB; investigation and formal analysis: SK, JPT, PFH and WB; writing - original draft: SK and WB; writing - review and editing: SK, KA, ASH, AL, MS, AH and MJB.

### Acknowledgments

The presented results are part of the doctoral thesis of Mr. Jannis Patrik Trier and Mrs. Pauline Friederike Heinzmann.

### Funding

The research work was conducted as a part of “Excellent Researchers in Breast Cancer” research project and was supported by Novartis Pharma GmbH Germany.

## Supplementary Material

Supplementary data
