# Supplementary material for: Anti-tumor effects of low-dose metronomic vinorelbine in combination with alpelisib in breast cancer cells
Source: EXCLI J. 2023 Jan 13;22:114–30. doi: 10.17179/excli2022-5064 (PMC10043427; doi:10.17179/excli2022-5064)

EXCLI Journal 2023;22:114-130 – ISSN 1611-2156
Received: June 12, 2022, accepted: January 12, 2023, published: January 13, 2023

Supplementary data to:

Original article:

ANTI-TUMOR EFFECTS OF LOW-DOSE METRONOMIC VINORELBINE IN COMBINATION WITH ALPELISIB IN BREAST CANCER CELLS

Slavomir Krajnak*, Jannis Patrik Trier, Pauline Friederike Heinzmann, Katharina Anic, Anne-Sophie Heimes, Amelie Loewe, Marcus Schmidt, Marco Johannes Battista, Annette Hasenburg, Walburgis Brenner

Clinic for Obstetrics and Women's Health, University Medical Center of the Johannes Gutenberg-University Mainz, Mainz, Germany

* **Corresponding author:** Dr. med. Slavomir Krajnak, Clinic for Obstetrics and Women's Health, University Medical Center of the Johannes Gutenberg-University Mainz, Langenbeckstraße 1, 55131 Mainz, Germany. Telephone: +49 (0) 6131-17-0; E-mail: slavomir.krajnak@unimedizin-mainz.de

https://dx.doi.org/10.17179/excli2022-5064

This is an Open Access article distributed under the terms of the Creative Commons Attribution License (http://creativecommons.org/licenses/by/4.0/).

EXCLI Journal 2023;22:114-130 – ISSN 1611-2156
Received: June 12, 2022, accepted: January 12, 2023, published: January 13, 2023

Raw data of Figure 1a

Cell viability of MCF-7 cells exposed to low-dose vinorelbine and alpelisib for 3 days. The data given are absolute values obtained from the Alamar blue assay.

Experiment 1		alpelisib (ng/ml)				
		0	10	100	500	1000
vinorelbine (ng/ml)	0	148668	127258	98637	78993	45059
	0.63	108262	92389	72531	57246	40065
	1.25	99206	85242	60447	52307	38667
	2.5	71181	50403	45840	47722	44585
	5	68580	52565	47001	48136	48268

Experiment 2		alpelisib (ng/ml)				
		0	10	100	500	1000
vinorelbine (ng/ml)	0	133536	105016	73586	51194	37501
	0.63	125618	90354	65902	51628	33269
	1.25	94497	67790	50800	44329	34833
	2.5	62858	41916	42220	40810	35639
	5	49127	40270	39377	39819	29834

Experiment 3		alpelisib (ng/ml)				
		0	10	100	500	1000
vinorelbine (ng/ml)	0	148898	133075	99682	66300	42274
	0.63	137366	103852	77789	58460	42404
	1.25	104655	76010	67604	54070	46259
	2.5	70703	51761	53993	48133	39621
	5	55536	49099	51117	43122	37460

Experiment 1-3 (mean values)		alpelisib (ng/ml)				
		0	10	100	500	1000
vinorelbine (ng/ml)	0	143701	121783	90635	65495	41611
	0.63	123749	95531	72074	55778	38579
	1.25	99453	76347	59617	50235	39920
	2.5	68247	48026	47351	45555	39948
	5	57748	47311	45832	43692	38520

EXCLI Journal 2023;22:114-130 – ISSN 1611-2156
Received: June 12, 2022, accepted: January 12, 2023, published: January 13, 2023

Raw data of Figure 1b

Cell viability of MCF-7 cells exposed to low-dose vinorelbine and alpelisib for 7 days. The data given are absolute values obtained from the Alamar blue assay.

Experiment 1		alpelisib (ng/ml)				
		0	10	100	500	1000
vinorelbine (ng/ml)	0	171932	156772	134224	85336	54213
	0.63	156575	123534	105861	60235	38198
	1.25	164582	129838	69975	47179	35518
	2.5	96617	46486	34033	32860	33749
	5	64818	45068	33786	32744	32439

Experiment 2		alpelisib (ng/ml)				
		0	10	100	500	1000
vinorelbine (ng/ml)	0	204800	197285	146623	82155	36443
	0.63	209299	208887	117180	51994	33200
	1.25	201375	115199	69389	45259	32324
	2.5	75709	34515	35076	29220	27099
	5	43697	33893	30765	26365	23758

Experiment 3		alpelisib (ng/ml)				
		0	10	100	500	1000
vinorelbine (ng/ml)	0	195832	186462	134042	58626	33756
	0.63	199710	163641	93616	50416	29143
	1.25	164638	84324	52641	37762	28448
	2.5	57891	28548	27091	30085	27146
	5	34683	25675	25849	28169	20854

Experiment 1-3 (mean values)		alpelisib (ng/ml)				
		0	10	100	500	1000
vinorelbine (ng/ml)	0	190855	180173	138296	75372	41470
	0.63	188528	165354	105552	54215	33513
	1.25	176865	109787	64002	43400	32097
	2.5	76739	36516	32067	30722	29331
	5	47733	34879	30133	29093	25684

EXCLI Journal 2023;22:114-130 – ISSN 1611-2156
Received: June 12, 2022, accepted: January 12, 2023, published: January 13, 2023

Raw data of Figure 1c

Cell viability of T-47D cells exposed to low-dose vinorelbine and alpelisib for 3 days. The data given are absolute values obtained from the Alamar blue assay.

Experiment 1		alpelisib (ng/ml)				
		0	10	100	500	1000
vinorelbine (ng/ml)	0	80389	54281	39122	33450	28740
	0.63	76222	56864	39836	34551	29530
	1.25	74489	54745	40869	35331	30123
	2.5	53461	45779	38521	34973	31955
	5	45595	39346	36097	34367	31291

Experiment 2		alpelisib (ng/ml)				
		0	10	100	500	1000
vinorelbine (ng/ml)	0	84547	69144	45773	40146	27949
	0.63	84464	66535	42006	38239	29226
	1.25	55808	50958	41849	38975	30996
	2.5	45524	44090	38620	35257	33670
	5	43481	41802	36906	34001	30155

Experiment 3		alpelisib (ng/ml)				
		0	10	100	500	1000
vinorelbine (ng/ml)	0	127768	107576	69295	59928	45274
	0.63	120912	102626	61763	57750	50231
	1.25	101499	101495	66512	58218	55567
	2.5	103714	114516	63282	57917	56769
	5	69663	62957	53792	57066	46315

Experiment 1-3 (mean values)		alpelisib (ng/ml)				
		0	10	100	500	1000
vinorelbine (ng/ml)	0	97568	77000	51396	44508	33988
	0.63	93866	75342	47868	43513	36329
	1.25	77265	69066	49743	44175	38895
	2.5	67566	68128	46807	42716	40798
	5	52913	48035	42265	41812	35920

Raw data of Figure 1d

Cell viability of T-47D cells exposed to low-dose vinorelbine and alpelisib for 7 days. The data given are absolute values obtained from the Alamar blue assay.

Experiment 1		alpelisib (ng/ml)				
		0	10	100	500	1000
vinorelbine (ng/ml)	0	193690	131996	51309	27559	21846
	0.63	161513	110920	45479	28850	18252
	1.25	173616	112467	45314	31685	19743
	2.5	135873	89099	38984	25025	22549
	5	50537	38569	26404	18724	18062

Experiment 2		alpelisib (ng/ml)				
		0	10	100	500	1000
vinorelbine (ng/ml)	0	167688	131956	51883	34554	22905
	0.63	161751	123510	49647	30445	21829
	1.25	91103	67587	39027	29991	19089
	2.5	50049	40309	26174	22127	18561
	5	37354	36365	26819	21836	18907

Experiment 3		alpelisib (ng/ml)				
		0	10	100	500	1000
vinorelbine (ng/ml)	0	169330	101905	44061	27552	19827
	0.63	159637	101720	41889	26309	19384
	1.25	157112	97994	39935	25411	20112
	2.5	55863	40307	30716	22301	18945
	5	33525	30204	25583	20758	19469

Experiment 1-3 (mean values)		alpelisib (ng/ml)				
		0	10	100	500	1000
vinorelbine (ng/ml)	0	176903	121952	49084	29888	21526
	0.63	160967	112050	45672	28535	19821
	1.25	140610	92683	41425	29029	19648
	2.5	80595	56571	31958	23151	20018
	5	40472	35046	26268	20439	18813

EXCLI Journal 2023;22:114-130 – ISSN 1611-2156
Received: June 12, 2022, accepted: January 12, 2023, published: January 13, 2023

Raw data of Figure 1e

Cell viability of MDA-MB-231 cells exposed to low-dose vinorelbine and alpelisib for 3 days. The data given are absolute values obtained from the Alamar blue assay.

Experiment 1		alpelisib (ng/ml)				
		0	10	100	500	1000
vinorelbine (ng/ml)	0	61794	59868	61311	58838	40175
	0.63	39868	43899	43959	43084	34894
	1.25	33504	37476	40291	38419	34247
	2.5	31014	34084	37016	35147	32462
	5	30141	30392	32607	33794	33967

Experiment 2		alpelisib (ng/ml)				
		0	10	100	500	1000
vinorelbine (ng/ml)	0	61037	61061	62279	57921	43499
	0.63	37151	37972	41660	47730	34360
	1.25	35468	37574	36375	37044	32643
	2.5	32123	33268	33970	34072	30658
	5	32022	36058	35564	33582	27235

Experiment 3		alpelisib (ng/ml)				
		0	10	100	500	1000
vinorelbine (ng/ml)	0	93998	96690	91967	82006	63384
	0.63	85000	88959	83211	77337	66273
	1.25	72208	75375	74242	68481	61305
	2.5	58716	63804	64542	58519	48429
	5	49333	52239	51685	38954	48054

Experiment 1-3 (mean values)		alpelisib (ng/ml)				
		0	10	100	500	1000
vinorelbine (ng/ml)	0	72276	72540	71852	66255	49019
	0.63	54006	56943	56276	56050	45176
	1.25	47060	50142	50303	47981	42732
	2.5	40618	43719	45176	42579	37183
	5	37165	39563	39952	35444	36419

EXCLI Journal 2023;22:114-130 – ISSN 1611-2156
Received: June 12, 2022, accepted: January 12, 2023, published: January 13, 2023

Raw data of Figure 1f

Cell viability of MDA-MB-231 cells exposed to low-dose vinorelbine and alpelisib for 7 days. The data given are absolute values obtained from the Alamar blue assay.

Experiment 1		alpelisib (ng/ml)				
		0	10	100	500	1000
vinorelbine (ng/ml)	0	195966	204573	212730	203997	154582
	0.63	167591	183610	189797	168018	106697
	1.25	112138	110248	111482	107902	82419
	2.5	75626	61779	75674	61911	61668
	5	46311	47854	49769	45158	32459

Experiment 2		alpelisib (ng/ml)				
		0	10	100	500	1000
vinorelbine (ng/ml)	0	190415	201873	209408	188710	151086
	0.63	179303	191257	186129	167446	131928
	1.25	123112	127872	124523	104987	85195
	2.5	65213	71850	69369	57994	45406
	5	45761	48902	47174	32827	37163

Experiment 3		alpelisib (ng/ml)				
		0	10	100	500	1000
vinorelbine (ng/ml)	0	175990	195792	193227	198392	128460
	0.63	164006	180300	178531	168474	141094
	1.25	144395	168927	165489	144328	115899
	2.5	106946	108916	108633	82607	69580
	5	48785	52832	57724	59829	41226

Experiment 1-3 (mean values)		alpelisib (ng/ml)				
		0	10	100	500	1000
vinorelbine (ng/ml)	0	187457	200746	205122	197033	144709
	0.63	170300	185056	184819	167980	126573
	1.25	126548	135682	133831	119072	94504
	2.5	82595	80848	84559	67504	58885
	5	46953	49863	51556	45938	36949

EXCLI Journal 2023;22:114-130 – ISSN 1611-2156
Received: June 12, 2022, accepted: January 12, 2023, published: January 13, 2023

Raw data of Figure 1g

Cell viability of BT-549 cells exposed to low-dose vinorelbine and alpelisib for 3 days. The data given are absolute values obtained from the Alamar blue assay.

Experiment 1		alpelisib (ng/ml)				
		0	10	100	500	1000
vinorelbine (ng/ml)	0	122969	127639	132337	126233	76575
	0.63	72023	66963	74615	61329	58033
	1.25	50633	53011	56771	54179	45636
	2.5	42724	43778	43978	43596	40941
	5	35876	37301	38292	38376	35539

Experiment 2		alpelisib (ng/ml)				
		0	10	100	500	1000
vinorelbine (ng/ml)	0	129870	151122	144121	143063	103378
	0.63	79769	86351	92035	89909	68273
	1.25	48503	52903	53321	45737	40684
	2.5	31541	33365	32979	28748	23173
	5	26544	29447	30601	28361	23593

Experiment 3		alpelisib (ng/ml)				
		0	10	100	500	1000
vinorelbine (ng/ml)	0	184266	211858	185009	158958	133263
	0.63	163510	157768	181268	144048	137693
	1.25	159815	170216	187129	165647	135804
	2.5	120090	124971	134726	114968	105457
	5	102617	89677	96710	83652	66308

Experiment 1-3 (mean values)		alpelisib (ng/ml)				
		0	10	100	500	1000
vinorelbine (ng/ml)	0	145702	163540	153822	142751	104405
	0.63	105101	103694	115973	98429	88000
	1.25	86317	92043	99074	88521	74041
	2.5	64785	67371	70561	62437	56524
	5	55012	52142	55201	50129	41813

EXCLI Journal 2023;22:114-130 – ISSN 1611-2156
Received: June 12, 2022, accepted: January 12, 2023, published: January 13, 2023

Raw data of Figure 1h

Cell viability of BT-549 cells exposed to low-dose vinorelbine and alpelisib for 7 days. The data given are absolute values obtained from the Alamar blue assay.

Experiment 1		alpelisib (ng/ml)				
		0	10	100	500	1000
vinorelbine (ng/ml)	0	158984	154175	108083	90233	100676
	0.63	84761	59123	61739	52318	57790
	1.25	36897	35188	34308	26389	28957
	2.5	37911	29393	28732	25951	21070
	5	28764	26160	24587	21236	20226

Experiment 2		alpelisib (ng/ml)				
		0	10	100	500	1000
vinorelbine (ng/ml)	0	193204	179814	203655	210063	163677
	0.63	166794	157263	171579	179024	150049
	1.25	89259	84116	82188	69896	60921
	2.5	40928	39740	37676	29052	22270
	5	28248	28263	29094	28376	20600

Experiment 3		alpelisib (ng/ml)				
		0	10	100	500	1000
vinorelbine (ng/ml)	0	129337	135434	147294	100354	85077
	0.63	125765	110994	106990	85354	77476
	1.25	121614	118453	103949	87382	82366
	2.5	89442	67359	76507	61516	59760
	5	45774	41367	47238	37882	28752

Experiment 1-3 (mean values)		alpelisib (ng/ml)				
		0	10	100	500	1000
vinorelbine (ng/ml)	0	160508	156474	153011	133550	116477
	0.63	125773	109127	113436	105565	95105
	1.25	82590	79252	73482	61223	57415
	2.5	56094	45497	47638	38839	34367
	5	34262	31930	33640	29165	23193

EXCLI Journal 2023;22:114-130 – ISSN 1611-2156
Received: June 12, 2022, accepted: January 12, 2023, published: January 13, 2023

Raw data of Figure 2

Cell viability of MCF-7, T-47D, MDA-MB-231 and BT-549 cells exposed to DMSO for 7 days. The data given are absolute values obtained from the Alamar blue assay.

Cell line	Experiment	Substance		
		0 ng/ml vinorelbine + 0 ng/ml alpelisib	DMSO 0.1%	DMSO 0.5%
MCF-7	1	184532	192998	170144
	2	181931	195710	196777
	3	181193	194407	189372
	1-3 (mean values)	182552	194371	185431
T-47D	1	139899	158407	146739
	2	185764	163862	152274
	3	156171	132159	112620
	1-3 (mean values)	160611	151476	137211
MDA-MB-231	1	131439	122267	122628
	2	130203	149211	142962
	3	132887	173445	162459
	1-3 (mean values)	131509	148308	142683
BT-549	1	185384	206080	183498
	2	209282	160720	89839
	3	167656	162516	150301
	1-3 (mean values)	187441	176439	141213

EXCLI Journal 2023;22:114-130 – ISSN 1611-2156
Received: June 12, 2022, accepted: January 12, 2023, published: January 13, 2023

Raw data of Figure 3a

Cell proliferation of MCF-7 cells exposed to low-dose vinorelbine and alpelisib for 7 days. The data given are absolute values obtained from the BrdU incorporation.

Experiment 1		alpelisib (ng/ml)				
		0	10	100	500	1000
vinorelbine (ng/ml)	0	1.9547	1.8246	1.8524	1.5434	1.6440
	0.63	1.6787	1.7305	1.8334	1.5018	1.4586
	1.25	1.5766	1.6672	1.7479	1.4288	1.3178
	2.5	1.6983	1.3884	1.1516	1.2010	1.1747
	5	1.1547	0.8139	0.8457	1.2112	0.9549

Experiment 2		alpelisib (ng/ml)				
		0	10	100	500	1000
vinorelbine (ng/ml)	0	2.3171	1.7432	0.4977	0.3298	0.2509
	0.63	0.4851	0.3175	0.1569	0.1822	0.1601
	1.25	0.6422	0.5037	0.4448	0.5569	0.3257
	2.5	0.5891	0.4870	0.6225	0.6065	0.3526
	5	0.4874	0.3758	0.3171	0.2091	0.2053

Experiment 3		alpelisib (ng/ml)				
		0	10	100	500	1000
vinorelbine (ng/ml)	0	2.0304	1.9776	1.5397	1.0369	0.7923
	0.63	1.6517	1.6548	1.3505	1.1765	0.6792
	1.25	1.9432	1.8095	1.2957	0.9525	0.8086
	2.5	1.4516	1.2976	0.9524	0.8730	0.9773
	5	1.0310	0.7616	0.5558	0.5456	0.6051

Experiment 1-3 (mean values)		alpelisib (ng/ml)				
		0	10	100	500	1000
vinorelbine (ng/ml)	0	2.1007	1.8485	1.2966	0.9700	0.8957
	0.63	1.2718	1.2342	1.1136	0.9535	0.7659
	1.25	1.3873	1.3268	1.1628	0.9794	0.8174
	2.5	1.2463	1.0577	0.9089	0.8935	0.8348
	5	0.8910	0.6504	0.5729	0.6553	0.5884

Raw data of Figure 3b

Cell proliferation of T-47D cells exposed to low-dose vinorelbine and alpelisib for 7 days. The data given are absolute values obtained from the BrdU incorporation.

Experiment 1		alpelisib (ng/ml)				
		0	10	100	500	1000
vinorelbine (ng/ml)	0	2.1488	1.5954	0.4569	0.2234	0.1949
	0.63	1.4878	0.8599	0.2967	0.1855	0.2244
	1.25	0.6780	0.4150	0.3657	0.2220	0.1769
	2.5	0.3233	0.2496	0.2786	0.5723	0.3230
	5	0.5095	0.2407	0.2174	0.2276	0.2356

Experiment 2		alpelisib (ng/ml)				
		0	10	100	500	1000
vinorelbine (ng/ml)	0	2.0172	1.3280	0.5255	0.5771	0.3000
	0.63	1.1713	0.5776	0.3812	0.4877	0.1893
	1.25	0.4152	0.3466	0.3558	0.4606	0.3138
	2.5	0.4545	0.3190	0.4753	1.0658	0.3867
	5	0.4921	0.3289	0.3430	0.3938	0.3381

Experiment 3		alpelisib (ng/ml)				
		0	10	100	500	1000
vinorelbine (ng/ml)	0	1.9065	1.1586	0.3399	0.2667	0.5549
	0.63	0.9999	0.4999	0.2851	0.6307	0.2107
	1.25	0.3165	0.2233	0.1251	0.2070	0.2055
	2.5	0.4558	0.2514	0.2036	0.2476	0.2821
	5	0.5892	0.2594	0.3108	0.4386	0.5346

Experiment 1-3 (mean values)		alpelisib (ng/ml)				
		0	10	100	500	1000
vinorelbine (ng/ml)	0	2.0242	1.3606	0.4408	0.3557	0.3499
	0.63	1.2197	0.6458	0.3210	0.4346	0.2081
	1.25	0.4699	0.3283	0.2822	0.2965	0.2321
	2.5	0.4112	0.2733	0.3192	0.6286	0.3306
	5	0.5303	0.2763	0.2904	0.3533	0.3694

Received: June 12, 2022, accepted: January 12, 2023, published: January 13, 2023

Raw data of Figure 3c

Cell proliferation of MDA-MB-231 cells exposed to low-dose vinorelbine and alpelisib for 7 days. The data given are absolute values obtained from the BrdU incorporation.

Experiment 1		alpelisib (ng/ml)				
		0	10	100	500	1000
vinorelbine (ng/ml)	0	2.5314	2.4398	2.4106	2.3118	2.5132
	0.63	2.4882	2.3020	2.2830	2.0875	2.0943
	1.25	1.8035	1.5327	1.5152	1.5290	1.3276
	2.5	1.0104	0.9060	0.7713	1.0491	0.8719
	5	0.7420	0.8973	0.6146	0.8218	0.6292

Experiment 2		alpelisib (ng/ml)				
		0	10	100	500	1000
vinorelbine (ng/ml)	0	1.9956	1.9361	1.8266	1.6027	1.7766
	0.63	1.9436	1.9663	1.9324	1.5741	1.7226
	1.25	2.0509	1.7878	1.6260	1.3611	1.5729
	2.5	1.2141	1.2033	1.4266	1.0698	0.9349
	5	0.9114	0.9008	0.8981	0.7107	0.9505

Experiment 3		alpelisib (ng/ml)				
		0	10	100	500	1000
vinorelbine (ng/ml)	0	2.5043	2.3611	2.3384	2.3247	2.5091
	0.63	2.1935	1.9351	1.8294	2.1522	1.7946
	1.25	1.4151	1.3649	1.1668	1.3357	1.1150
	2.5	0.8641	0.8600	0.7544	0.8227	0.7243
	5	0.6786	0.6370	0.5966	0.6937	0.5682

Experiment 1-3 (mean values)		alpelisib (ng/ml)				
		0	10	100	500	1000
vinorelbine (ng/ml)	0	2.3438	2.2457	2.1919	2.0797	2.2663
	0.63	2.2084	2.0678	2.0149	1.9379	1.8705
	1.25	1.7565	1.5618	1.4360	1.4086	1.3385
	2.5	1.0295	0.9898	0.9841	0.9806	0.8437
	5	0.7773	0.8117	0.7031	0.7421	0.7160

EXCLI Journal 2023;22:114-130 – ISSN 1611-2156
Received: June 12, 2022, accepted: January 12, 2023, published: January 13, 2023

Raw data of Figure 4a-4d

Cell viability of MCF-7, T-47D, MDA-MB-231 and BT-549 cells exposed to low-dose vinorelbine and alpelisib alone for 3 and 7 days. The data are shown as percentages relative to those of the untreated control cell culture. The absolute values of the Alamar blue assay are displayed in the tables of raw data of Figure 1.

Duration of treatment (days)	Cell line	Substance				
		vinorelbine (ng/ml)				
		0	0.63	1.25	2.5	5
3	MCF-7	100	86.1	69.2	47.5	40.2
	T-47D	100	96.2	79.2	69.3	54.2
	MDA-MB-231	100	74.7	65.1	56.2	51.4
	BT-549	100	72.1	59.2	44.5	37.8
7	MCF-7	100	98.8	92.7	40.2	25.0
	T-47D	100	91.0	79.5	45.6	22.9
	MDA-MB-231	100	90.8	67.5	44.1	25.0
	BT-549	100	78.4	51.5	34.9	21.3
		alpelisib (ng/ml)				
		0	10	100	500	1000
3	MCF-7	100	84.7	63.1	45.6	29.0
	T-47D	100	78.9	52.7	45.6	34.8
	MDA-MB-231	100	100.4	99.4	91.7	67.8
	BT-549	100	112.2	105.6	98.0	71.7
7	MCF-7	100	94.4	72.5	39.5	21.7
	T-47D	100	68.9	27.7	16.9	12.2
	MDA-MB-231	100	107.1	109.4	105.1	77.2
	BT-549	100	97.5	95.3	83.2	72.6

EXCLI Journal 2023;22:114-130 – ISSN 1611-2156
Received: June 12, 2022, accepted: January 12, 2023, published: January 13, 2023

Raw data of Figure 4e and 4f

Cell proliferation of MCF-7, T-47D and MDA-MB-231 cells exposed to low-dose vinorelbine and alpelisib alone for 7 days. The data are shown as percentages relative to those of the untreated control cell culture. The absolute values of the BrdU incorporation are displayed in the tables of raw data of Figure 3.

Cell line	Substance				
	vinorelbine (ng/ml)				
	0	0.63	1.25	2.5	5
MCF-7	100	60.5	66.0	59.3	42.4
T-47D	100	60.3	23.2	20.3	26.2
MDA-MB-231	100	94.2	74.9	43.9	33.2
	alpelisib (ng/ml)				
	0	10	100	500	1000
MCF-7	100	88.0	61.7	46.2	42.6
T-47D	100	67.2	21.8	17.6	17.3
MDA-MB-231	100	95.8	93.5	88.7	96.7

EXCLI Journal 2023;22:114-130 – ISSN 1611-2156
Received: June 12, 2022, accepted: January 12, 2023, published: January 13, 2023

Raw data of Figure 6a

The p110α expression in the MCF-7 and T-47D cells after 7 days of treatment with low-dose vinorelbine plus alpelisib was quantified by densitometry evaluation using a computer-based pixel counting system (AlphaView, ProteinSimple, San Jose, CA, USA). These values were referenced to the β-actin values of the same membrane as a loading control.

Cell line	Substance		Experiment		
	vinorelbine (ng/ml)	alpelisib (ng/ml)	1	2	3
MCF-7	0	0	1	1	1
	0	10	0.4413	0.8242	0.9194
	0	100	0.8784	0.8747	0.7108
	0.63	0	0.8787	1.0189	0.9133
	0.63	10	0.9571	0.5421	0.8919
	0.63	100	0.7847	0.5926	0.5831
	1.25	0	0.7620	0.3128	0.6713
	1.25	10	0.9243	0.2228	0.6798
	1.25	100	1.2537	0.9955	0.6408
	2.5	0	1.0444	1.3702	0.8552
	2.5	10	0.9887	1.0692	1.1308
	2.5	100	1.1004	0.7936	1.1793
T-47D	0	0	1	1	1
	0	10	0.6534	0.8784	0.4548
	0	100	0.7675	0.6511	0.4505
	0.63	0	0.8421	1.0687	0.5459
	0.63	10	0.4940	0.7981	0.4686
	0.63	100	0.3927	0.5763	0.5427
	1.25	0	0.7177	1.0457	0.7822
	1.25	10	0.5102	0.7895	0.5499
	1.25	100	0.3720	0.7084	0.4337
	2.5	0	0.4106	1.5800	0.5987
	2.5	10	0.4668	1.0556	0.4192
	2.5	100	0.4313	0.4355	0.6202

EXCLI Journal 2023;22:114-130 – ISSN 1611-2156
Received: June 12, 2022, accepted: January 12, 2023, published: January 13, 2023

Raw images of the Western blot analyses for the p110α and β-actin protein in the MCF-7 cells (Figure 6a)

Experiment 1

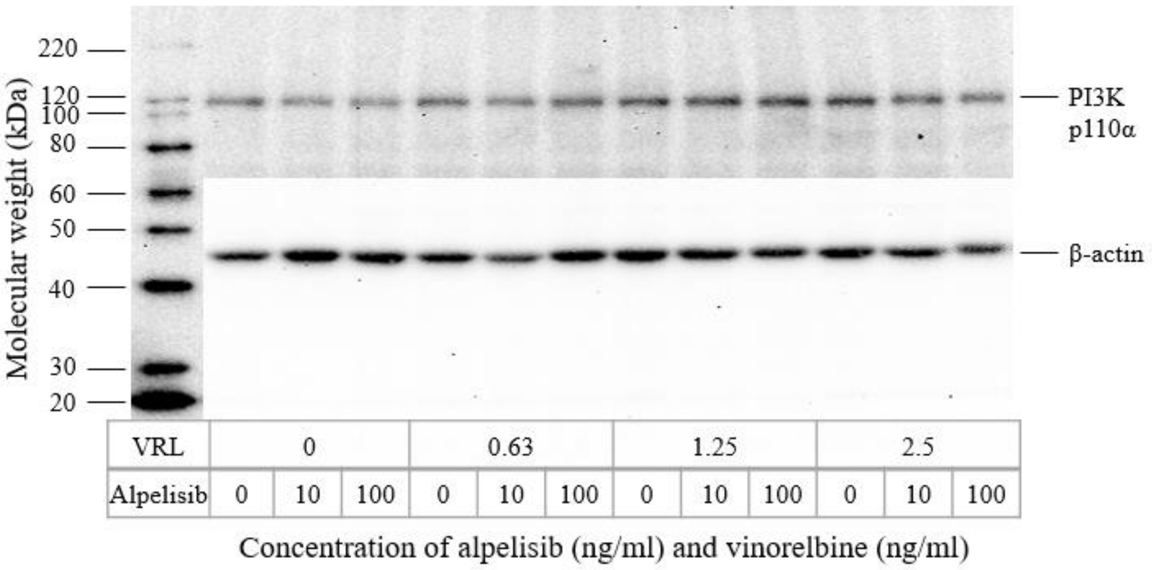

Experiment 2

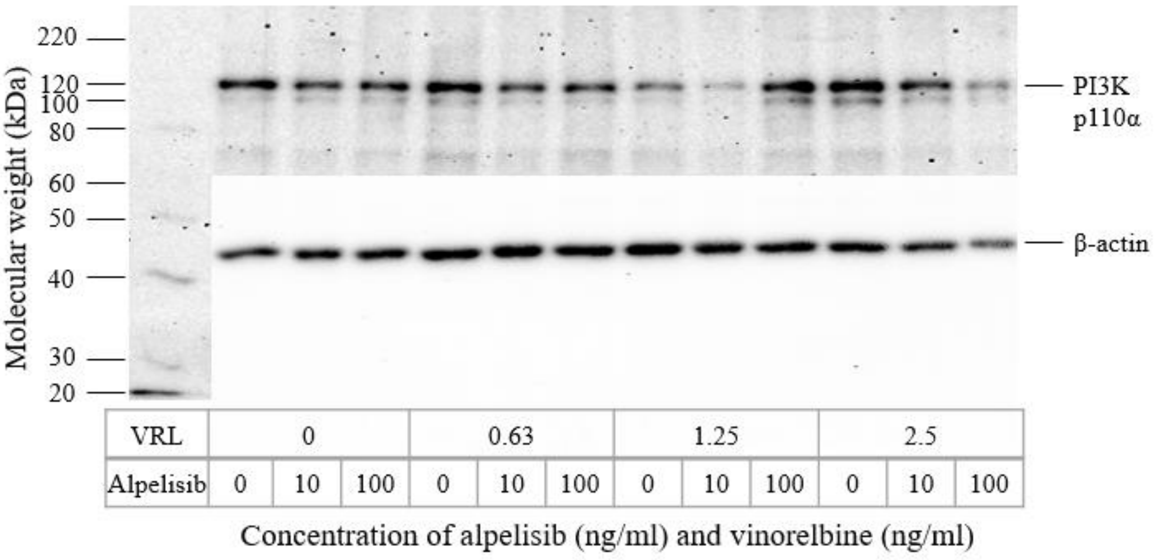

Experiment 3

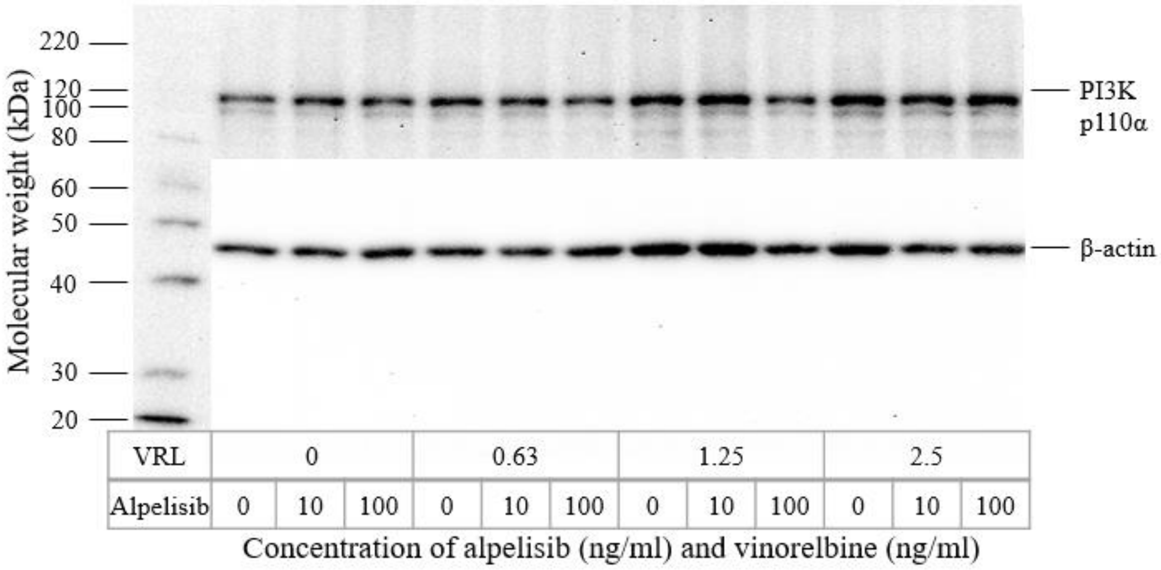

Raw images of the Western blot analyses for the p110α and β-actin protein in the T-47D cells (Figure 6a)

Experiment 1

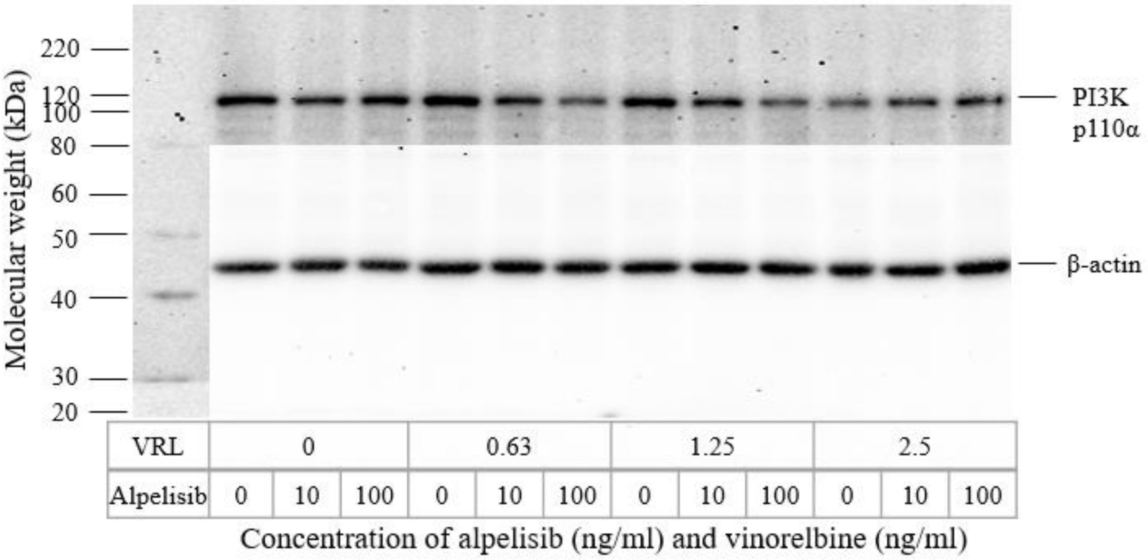

EXCLI Journal 2023;22:114-130 – ISSN 1611-2156
Received: June 12, 2022, accepted: January 12, 2023, published: January 13, 2023

Experiment 2

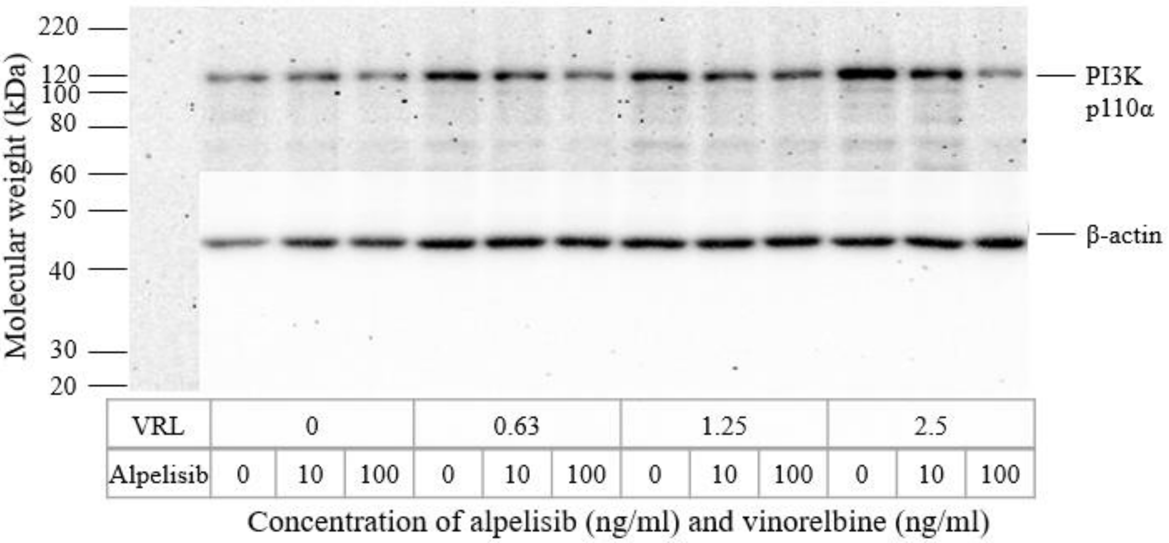

Experiment 3

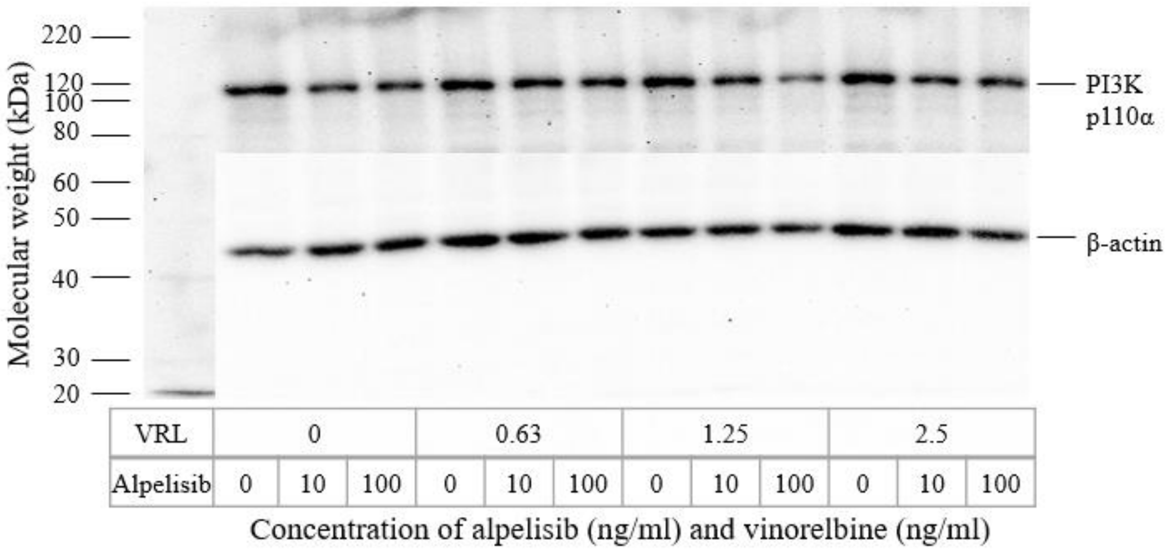

EXCLI Journal 2023;22:114-130 – ISSN 1611-2156
Received: June 12, 2022, accepted: January 12, 2023, published: January 13, 2023

Raw data of Figure 6b

The p110α expression in the MDA-MB-231 and BT-549 cells after 7 days of treatment with low-dose vinorelbine plus alpelisib was quantified by densitometry evaluation using a computer-based pixel counting system (AlphaView, ProteinSimple, San Jose, CA, USA). These values were referenced to the β-actin values of the same membrane as a loading control.

Cell line	Substance		Experiment		
	vinorelbine (ng/ml)	alpelisib (ng/ml)	1	2	3
MDA-MB-231	0	0	1	1	1
	0	10	0.9767	1.2853	1.0863
	0	100	0.9967	1.4718	1.1870
	0.63	0	0.8163	0.8542	0.8018
	0.63	10	1.0140	1.0273	0.9784
	0.63	100	1.1848	1.2281	1.0108
	1.25	0	0.8253	0.8100	0.7351
	1.25	10	0.8955	0.8434	0.5737
	1.25	100	0.9038	0.8688	0.8899
	2.5	0	0.7168	0.5802	0.5692
	2.5	10	0.9112	1.0041	0.5993
	2.5	100	0.8553	1.5883	1.1327
BT-549	0	0	1	1	1
	0	10	1.2357	1.0019	0.6863
	0	100	1.4895	1.0312	0.7612
	0.63	0	1.1639	0.6708	0.6248
	0.63	10	1.1381	0.9990	0.7823
	0.63	100	1.4911	1.2930	0.8440
	1.25	0	0.8677	1.0559	0.8341
	1.25	10	1.5531	0.8378	0.9114
	1.25	100	1.7476	1.2685	1.7279
	2.5	0	1.1202	1.3589	0.7969
	2.5	10	1.8483	1.0015	0.8232
	2.5	100	1.6525	1.4585	1.0358

EXCLI Journal 2023;22:114-130 – ISSN 1611-2156
Received: June 12, 2022, accepted: January 12, 2023, published: January 13, 2023

Raw images of the Western blot analyses for the p110α and β-actin protein in the MDA-MB-231 cells (Figure 6b)

Experiment 1

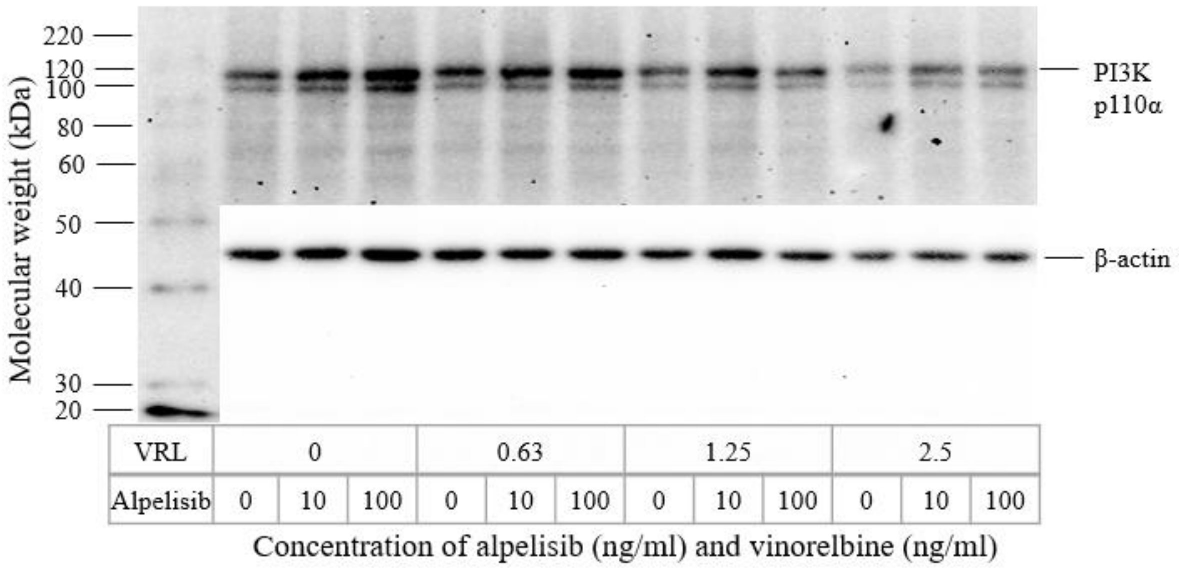

Experiment 2

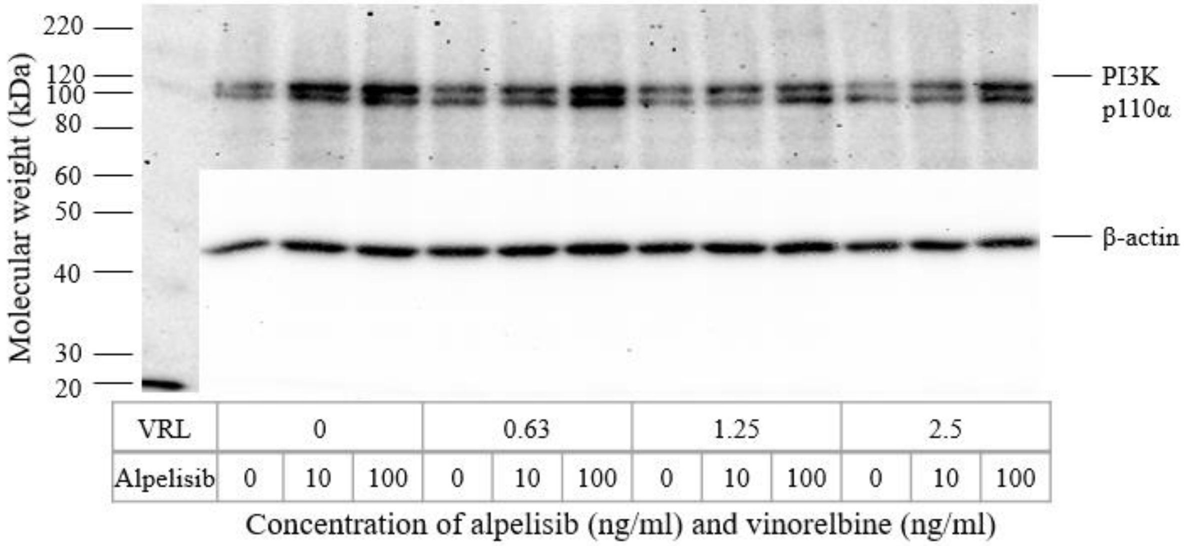

EXCLI Journal 2023;22:114-130 – ISSN 1611-2156
Received: June 12, 2022, accepted: January 12, 2023, published: January 13, 2023

Experiment 3

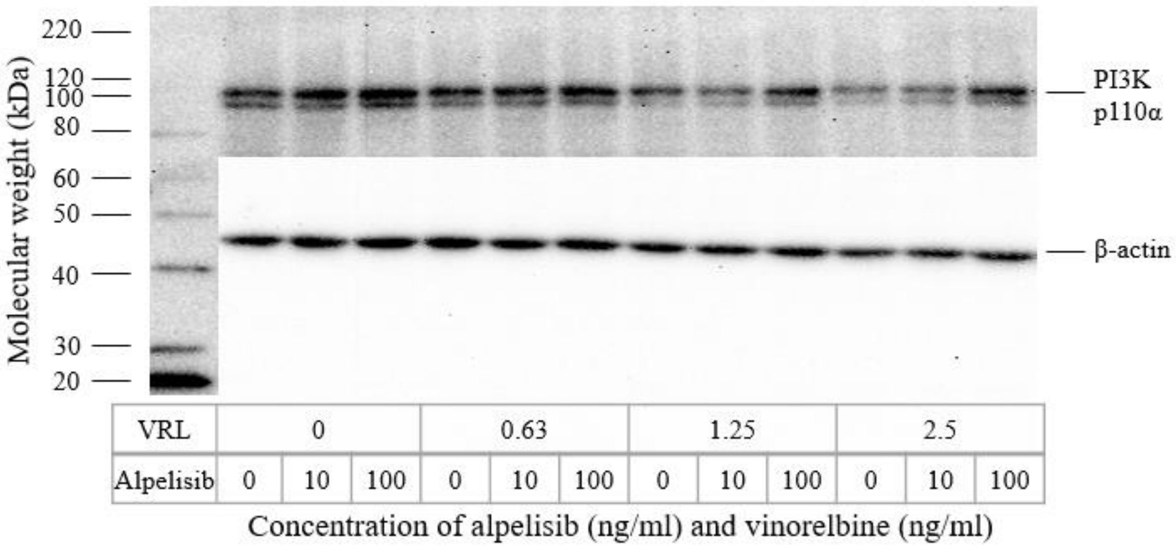

Raw images of the Western blot analyses for the p110α and β-actin protein in the BT-549 cells (Figure 6b)

Experiment 1

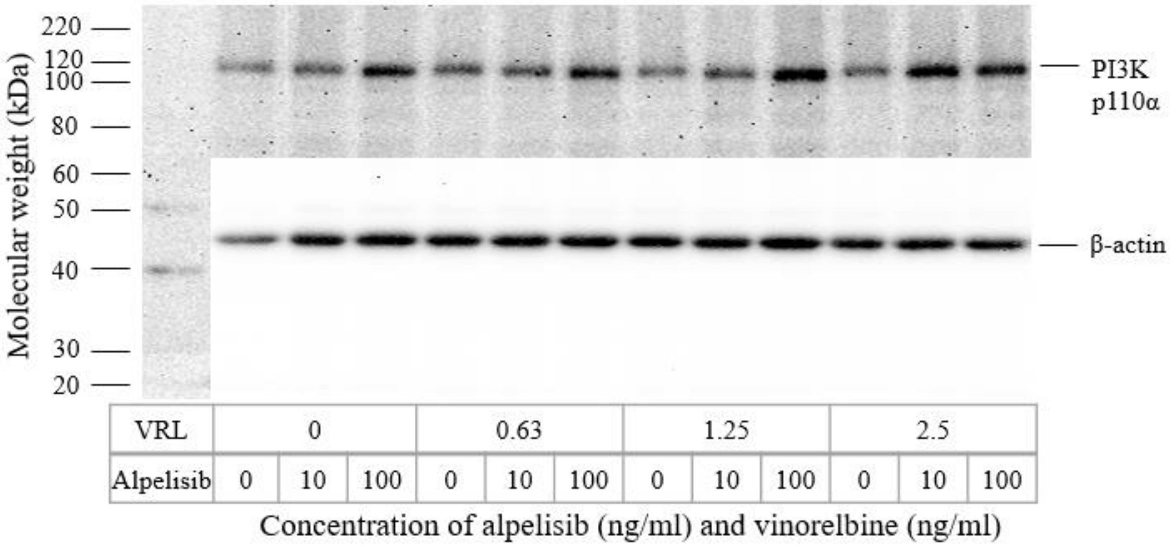

EXCLI Journal 2023;22:114-130 – ISSN 1611-2156
Received: June 12, 2022, accepted: January 12, 2023, published: January 13, 2023

Experiment 2

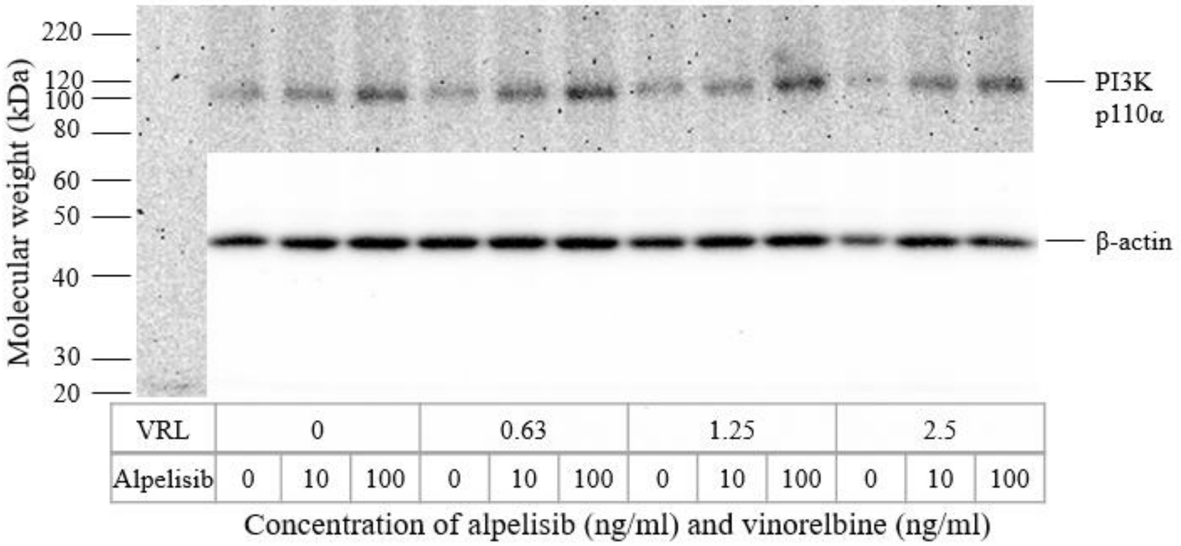

Experiment 3

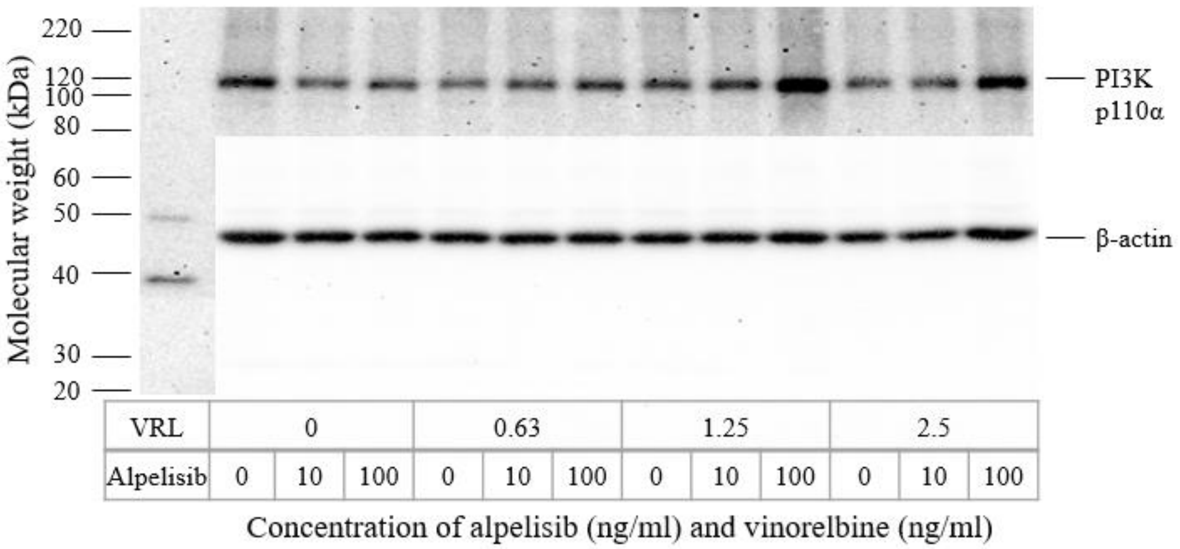

Supplement: Supplementary data [file EXCLI-22-114-s-001.pdf]
